# The association of genetic polymorphisms in *CYP1A2*, *UGT1A4*, and *ABCB1* with autonomic nervous system dysfunction in schizophrenia patients treated with olanzapine

**DOI:** 10.1186/s12888-020-02492-5

**Published:** 2020-02-18

**Authors:** Saki Hattori, Akira Suda, Masatoshi Miyauchi, Yohko Shiraishi, Takashi Saeki, Tadashi Fukushima, Mami Fujibayashi, Natsuki Tsujita, Chie Ishii, Norio Ishii, Tosiho Moritani, Yusuke Saigusa, Ikuko Kishida

**Affiliations:** 1 Department of Psychiatry, Yokohama City University School of Medicine, 3-9 Fukuura, Kanazawa-ku, Yokohama, Kanagawa 236-0004 Japan; 2 Asahinooka Hospital, 128-1 Kwaihonchou, Asahi-ku, Yokohama, Kanagawa 251-8530 Japan; 3 Division of Physical and Health Education, Setsunan University, 17-8 Ikedanakamachi, Neyagawa, Osaka, 572-8508 Japan; 4 Graduate School of Human and Environmental Studies, Kyoto University, Yoshidanihonmatsucho, Sakyo-ku, Kyoto, 606-8316 Japan; 5 Fujisawa Hospital, 383 Kotuka Fujisawa, Kanagawa, 251-8530 Japan; 6 Faculty of General Education, Kyoto Sangyo University, Kamo-motoyama, Kita-ku, Kyoto, 606-8555 Japan; 7 Department of Biostatistics, Yokohama City University School of Medicine, 3-9 Fukuura, Kanazawa-ku, Yokohama, Kanagawa 236-0004 Japan

**Keywords:** Schizophrenia, Olanzapine, Autonomic nervous system, UGT1A4, Gene polymorphism, Heart rate variability

## Abstract

**Background:**

Use of the antipsychotic drug olanzapine by patients with schizophrenia is associated with autonomic nervous system (ANS) dysfunction. It is presumed that there are interindividual differences in ANS dysfunction that correspond to pharmacogenetics. In this study, we investigated whether genetic polymorphisms in *ABCB1*, *CYP1A2*, and *UGT1A4* are associated with this observed ANS dysfunction.

**Methods:**

A total of 91 schizophrenia patients treated with olanzapine monotherapy participated in this study. A power spectral analysis of heart rate variability was used to assess ANS activity. The TaqMan system was used to genotype seven single nucleotide polymorphisms (SNPs) in *CYP1A2* (rs2069514 and rs762551), *UGT1A4* (rs2011425), and *ABCB1* (rs1045642, rs1128503, rs2032582, rs2235048).

**Results:**

Sympathetic nervous activity was significantly higher in individuals with the *UGT1A4* rs2011425 G allele than in those with the *UGT1A4* rs2011425 non-G allele (sympathetic activity, *p* = .001). Furthermore, sympathetic nervous activity was also significantly associated with *UGT1A4* rs2011425 genotype as revealed by multiple regression analysis (sympathetic activity, *p* = .008).

**Conclusions:**

We suggest that the *UGT1A4* rs2011425 polymorphism affects olanzapine tolerability because it is associated with the observed side effects of olanzapine in schizophrenia patients, namely sympathetic dysfunction.

## Background

Compared with the general population, patients with schizophrenia exhibit lower autonomic nervous system (ANS) activity [1]. Moreover, ANS dysfunction in schizophrenia patients is exacerbated by antipsychotic medications [2–4], and diminished ANS activity is associated with sudden death resulting from cardiovascular disease and overall morbidity [5]. We previously described the dose-dependent manner by which antipsychotic drugs significantly decrease ANS activity [4] and the dissimilar effects of respective atypical antipsychotics on ANS activity [6].

One of the most commonly used atypical antipsychotics, olanzapine, is prescribed worldwide, although its side effects include weight gain and glucose intolerance. We have reported that olanzapine appears to affect autonomic nervous activity strongly in comparison with other atypical antipsychotics such as risperidone or aripiprazole [6]. Furthermore, we have also demonstrated individual-level differences in ANS activity among olanzapine-treated patients [6]. Many studies have reported that genetics influence therapeutic outcomes [7] and that the side effects of antipsychotics such as olanzapine are affected by pharmacogenetics [8]. Moreover, a previous study has reported that the relevant polymorphisms in genes involved in olanzapine metabolism and bioavailability are related to patient responses to olanzapine [9].

Upon administration, olanzapine is broadly dispersed throughout the bodies of patients, with 93% of the compound binding to plasma proteins, such as albumin and alpha-1 acid glycoprotein. The liver extensively metabolizes olanzapine, primarily by direct glucuronidation and oxidation as mediated by the cytochrome p450 (CYP) isoenzyme CYP1A2 [10, 11]. Olanzapine is predominantly metabolized through glucuronidation by enzymes in the uridine 5′-diphospho-glucuronosyltransferase (UGT) protein family [10]. Additionally, P-glycoprotein (P-gp) is encoded by adenosine 5′′-triphosphate-binding cassette transport sub-family B member 1 (ABCB1), also known as multidrug resistance protein 1 (MDR1). P-gp both serves as an important efflux pump across the blood–brain barrier for olanzapine and influences olanzapine availability to the brain by transporting it through the blood–brain barrier against concentration gradients, thereby reducing its accumulation in brain tissue [12]. Accordingly, the absorption and distribution of olanzapine is substantially shaped by P-gp [13]. Associations between polymorphisms in *CYP1A2* and *ABCB1* sequences and treatment responses or weight gain have been previously reported [14, 15], and it is presumed that genetic polymorphisms that affect the serum concentration of olanzapine could be associated with ANS activity in olanzapine-treated patients with schizophrenia. However, no association studies examining these gene polymorphisms and ANS dysfunction resulting from olanzapine administration have been reported. Furthermore, although an effect of *UGT1A4* polymorphisms on olanzapine plasma levels has been reported [16], no published studies have characterized the impact of *UGT1A4* polymorphisms on side effects such as ANS dysfunction.

Thus, in the present study involving patients with schizophrenia, we investigated whether seven single nucleotide polymorphisms (SNPs) in *CYP1A2*, *UGT1A4*, and *ABCB1* are associated with side effects of olanzapine, including decreased ANS activity, to clarify the factors that affect interindividual differences in decreased ANS activity during olanzapine therapy and to provide safer medication that can be tailored to individual genotypes.

## Methods

### Participants

The present cross-sectional study was conducted with data collected from 91 Japanese patients diagnosed with schizophrenia (52 women and 39 men; age [mean ± standard deviation], 50.30 ± 15.25 years) under treatment at Asahinooka Hospital, Fujisawa Hospital, and Yokohama City University Hospital in Japan. Each of these patients was recruited on the basis of their schizophrenia diagnoses and their undergoing a stable olanzapine monotherapy for at least 3 months. Moreover, each of their antipsychotic treatments had been unchanged over the 3 months prior to this study. Participants were excluded if they were unable to take the antipsychotics as prescribed and/or had remained under hospital treatment for at least 1 year. Sufficiently experienced clinical psychiatrists diagnosed the patients according to the criteria described by the fourth edition of the *Diagnostic and Statistical Manual of Mental Disorders*. We further excluded participants who had been diagnosed with respiratory, cardiovascular, endocrine, or neurological ailments, were receiving medication to treat physical diseases, or exhibited a history of substance abuse that may have complicated schizophrenia diagnosis.

Symptom severity was assessed using a version of the Positive and Negative Syndrome Scale (PANSS) [17] that had been translated into Japanese. Accordingly, the positive, negative, and general signs of the patients were assessed by psychiatrists with sufficient clinical experience using the PANSS on the same day electrocardiography (ECG) recording was performed.

All psychotropic medications for each patient were assessed, including antiparkinsonian, benzodiazepine, and antipsychotic medications. Dosages of all patient medications were converted to standard equivalent units of biperiden, diazepam, and chlorpromazine [18].

The ethics committee of Fujisawa Hospital approved the study, which was conducted following the Declaration of Helsinki. All participants gave their informed consent following a full explanation of the present study.

### R–R interval power spectral analysis

As in our previous research [19, 20], a computer-assisted 5-min measurement of resting heart rate variability (HRV) was conducted to evaluate ANS activity. HRV power spectral analysis has been applied widely in basic medical science and clinical studies under various psychophysiological conditions and enables the effective noninvasive assessment of autonomic imbalances through the detection of the three major spectral components of HRV. Its reliability, practicability, and validity and have been repeatedly demonstrated previously [1, 21–26]. Each experimental session was conducted between 09:00 h and 12:00 h. Participants abstained from both nicotine and caffeine the morning before each measurement. ECG was performed with seated patients for 5 min after a resting period of at least 10 min. A fast Fourier transform was used in the HRV power spectral analysis to decompose a series of R–R intervals obtained from each 5-min ECG timeseries into sums of various sinusoidal functions of different frequencies and amplitudes [19, 20, 27, 28]. Following procedures from our previous studies [19, 20, 24, 25], spectral power was quantified for the frequency domain using estimates of the areas under the curve in three frequency bands: low-frequency (LF; 0.03–0.15 Hz) HRV, reflecting both parasympathetic and sympathetic nerve activity; high-frequency (HF; 0.15–0.40 Hz) HRV, primarily reflecting parasympathetic nerve activity; and total power (TP; 0.03–0.40 Hz), reflecting overall ANS activity [27, 28]. LF and HF work independently, and TP is the sum of LF and HF [29, 30]. A greater LF score indicates higher sympathetic activity. A greater HF score indicates higher parasympathetic activity, and a greater TP score indicates higher ANS activity. It is presumed that higher HRV is generally indicative of better health because previous studies have reported that lower HRV is associated with increased risk of death and cardiovascular disease [31, 32].

### DNA extraction, SNP selection, and genotyping

From each patient, a peripheral blood sample was collected for the purpose of DNA extraction and subsequent genotyping. Seven SNPs—rs2069514, rs762551, rs2011425, rs1045642, rs1128503, rs2032582, and rs2235048—were chosen from the National Center for Biotechnology Information dbSNP database (Table 1) on the basis of their potential effects on plasma olanzapine levels and/or olanzapine responses [14, 15, 33–35]. Following genomic DNA extraction from peripheral blood leukocytes, SNPs in *CYP1A2* (rs2069514, rs762551), *UGT1A4* (rs2011425), and *ABCB1* (rs1045642, rs1128503, rs2032582, rs2235048) were genotyped; these SNPs are summarized in Table 1. TaqMan SNP genotyping assays and the ABI Prism 7900HT sequence detection system (Applied Biosystems, Foster City, CA, USA) were used to genotype all SNPs.

**Table 1:** *CYP1A2*, *UGT1A4*, and *ABCB1* single nucleotide polymorphisms (SNPs)

Gene	SNP	Location	Alleles	Frequency (Global)	Frequency (Japanese)
*CYP1A2*	rs2069514	Chr.15: 74745879	G/A	0.209	0.236
	rs762551	Chr.15: 74749576	C/A	0.630	0.636
*UGT1A4*	rs2011425	Chr.2: 233718962	T/G	0.116	0.127
*ABCB1*	rs1045642	Chr.7: 87509329	C/T	0.503	0.577
	rs1128503	Chr.7: 87550285	C/T	0.542	0.384
	rs2032582	Chr.7: 87531302	G/A or T	0.543	0.422
	rs2235048	Chr.7: 87509195	T/C	0.602	0.585

Data from TogoVar [Internet]. Tokyo: Japan Science and Technology Agency (Japan), National Bioscience Database Center; 2020. Available from https://togovar.biosciencedbc.jp

### Statistical analyses

SPSS for Windows version 24 (IBM Corp., Armonk, NY, USA) was used to conduct all statistical analyses. Student’s *t*-tests were used to assess differences in the means of LF, HF, and TP HRV components between individuals bearing each of the alleles at each SNP. Bonferroni correction was applied for multiple comparisons: the Bonferroni-corrected critical *p*-value was 0.05/7 (i.e., *p* < 0.007). Multiple regression analysis was also employed to assess the effects of clinical factors on ANS activity, with LF, HF, and TP HRV components used as dependent variables, whereas the independent variables included age, body mass index, PANSS, dosages of antipsychotic, antiparkinsonian, and benzodiazepine agents, and genetic polymorphisms identified by the aforementioned Student’s *t*-tests as potentially affecting ANS activity [3, 36–38]. Student’s *t*-tests were also used to compare clinical and demographic data (i.e., age, body mass index [BMI], PANSS, duration of illness, and daily dosages of antipsychotic, antiparkinsonian, and benzodiazepine agents) between patients with the rs2011425 G and non-G alleles. Proportions of men and women and proportions of smokers and nonsmokers were examined using the chi-square test. Haploview version 4.1 was used to analyze linkage disequilibrium [39]. Absolute values of the HRV spectral components were log-transformed prior to analysis because these data were skewed. Significance was considered at a threshold of *p* < 0.05 except for comparisons of associations between genotypes and ANS activity.

## Results

Table 2 summarizes all demographic, medication, and HRV component data for all study participants. The daily dosage of each antipsychotic drug was converted to an approximate chlorpromazine equivalent (CPZ), with a patient mean of 491.21 ± 247.05 mg/day. The mean HRV components of individuals with various genotypes across the seven SNPs are summarized in Table 3. None of the SNPs exhibited a significant deviation from the expected Hardy–Weinberg proportions. Perfect linkage disequilibrium was revealed between *ABCB1* rs1045642 and rs2235048 by haplotype analysis (*r*^2^ = 1). The LF HRV component was significantly higher in the group with *UGT1A4* rs2011425 G alleles than that with non-G alleles (LF, *p* = .001; Table 3). The TP HRV component had a tendency to be higher in the group with *UGT1A4* rs2011425 G alleles than that with non-G alleles (*p* = .042; Table 3). The LF and TP HRV components also had a tendency to be lower among individuals with the *CYP1A2* rs762551 A allele relative to those with the non-A allele (LF, *p* = .013; TP, *p* = .020; Table 3). Multiple regression analysis revealed that the LF HRV component was significantly associated with the *UGT1A4* rs2011425 genotype (LF, *p* = .008; Table 4). In the multiple regression analysis, *CYP1A2* rs762551 genotype, BMI, PANSS score, dosages of antiparkinsonian agents, and dosages of benzodiazepine agents were not significantly associated with any HRV components. However, both LH and TP HRV components were significantly associated with age and dosages of antipsychotic compounds (Table 4). The medication and demographic data for patients with the *UGT1A4* rs2011425 G allele and non-G alleles are summarized in Table 5. However, there were no significant differences in sex, age, smoking status, BMI, total PANSS score, disease duration, or medication between carriers of the two alleles.

**Table 2:** Summary of patient demography and medication, with log-transformed power values of the LF, HF, and TP bands

	All subjects (*n* = 91)
Age (years)	50.30 ± 15.25
Sex (male/female)	39/52
Inpatient/outpatient	21/70
Duration of illness (years)	19.28 ± 13.35
Smoking (smoker/non-smoker)	6/85
BMI (kg/m^2^)	24.01 ± 4.66
CPZeq^a^ (mg/day)	491.21 ± 247.05
BPDeq^b^ (mg/day)	0.78 ± 1.79
DZPeq^c^ (mg/day)	7.62 ± 13.41
PANSS total score	72.16 ± 16.00
lnLF (ms^2^)	4.32 ± 1.12
lnHF (ms^2^)	3.81 ± 1.15
lnTotal Power (ms^2^)	4.93 ± 0.99

Data are presented as the mean ± standard deviation

*BMI* Body mass index, *PANSS* Positive and Negative Syndrome Scale, *ln* natural log-transformed, *HF* High frequency, *LF* Low frequency, *TP* Total power

^a^ The daily dosages of antipsychotic drugs were converted to approximate chlorpromazine equivalents

^b^ The daily dosages of anticholinergic antiparkinsonian drugs were converted to approximate biperiden equivalents

^c^ The daily dosages of benzodiazepine were converted to approximate diazepam equivalents

**Table 3:** Associations between *CYP1A2*, *UGT1A4*, and *ABCB1* genotypes and autonomic nervous system activity

*CYP1A2*						
	rs2069514			rs762551		
	G/G	G/A, A/A	*P*	C/C	C/A, A/A	*P*
	56	35		21	70	
lnLF (ms^2^)	4.40 ± 1.12	4.17 ± 1.11	0.337	4.84 ± 0.84	4.16 ± 1.15	0.013
lnHF (ms^2^)	3.86 ± 1.22	3.72 ± 1.05	0.597	4.15 ± 1.45	3.70 ± 1.03	0.119
lnTP (ms^2^)	4.98 ± 1.04	4.84 ± 0.92	0.511	5.37 ± 0.96	4.80 ± 0.97	0.020
*UGT1A4*						
	rs2011425					
	T/T	G/T, G/G	*P*			
	73	18				
lnLF (ms^2^)	4.14 ± 1.07	5.06 ± 1.04	0.001 ^a^			
lnHF (ms^2^)	3.59 ± 0.93	3.86 ± 1.20	0.378			
lnTP (ms^2^)	4.82 ± 0.98	5.35 ± 0.95	0.042			
*ABCB1*						
	rs1045642			rs1128503		
	C/C	C/T, T/T	*P*	T/T	C/T, C/C	*P*
	24	67		43	48	
lnLF (ms^2^)	4.27 ± 1.19	4.34 ± 1.10	0.803	4.26 ± 1.07	4.37 ± 1.17	0.649
lnHF (ms^2^)	3.97 ± 1.12	3.75 ± 1.16	0.423	3.69 ± 1.19	3.91 ± 1.12	0.370
lnTP (ms^2^)	4.96 ± 1.03	4.92 ± 0.98	0.845	4,85 ± 1.01	5.00 ± 0.98	0.486
*ABCB1*						
	rs2032582			rs2235048		
	G/G	G/A•T, A•T/A•T	*P*	T/T	C/C, C/T	*P*
	16	75		24	67	
lnLF (ms^2^)	4.11 ± 1.07	4.36 ± 1.13	0.419	4.27 ± 1.19	4.34 ± 1.10	0.803
lnHF (ms^2^)	3.80 ± 1.44	3.81 ± 1.09	0.977	3.97 ± 1.12	3.75 ± 1.16	0.423
lnTP (ms^2^)	4.83 ± 1.06	4.95 ± 0.98	0.665	4.96 ± 1.03	4.92 ± 0.98	0.845

Data are presented as mean ± standard deviation values

*ln* natural log-transformed, *HF* High frequency, *LF* Low frequency, *TP* Total power

^a^ Significant difference (*p* < 0.007; Student’s *t*-test)

**Table 4:** Multiple regression analysis results using ANS activity, age, sex, smoking status, BMI, PANSS, *CYP1A2* rs762551 genotype, and *UGT1A4* rs2011425 genotype as independent variables in participants treated with olanzapine monotherapy

Independent variable	ANS activity					
	lnLF		lnHF		lnTP	
	β	*P*	β	*P*	β	*P*
Age (years)	−0.027	< 0.001 ^d^	−0.002	0.804	−0.019	0.009 ^d^
BMI (kg/m^2^)	−0.020	0.367	−0.001	0.973	−0.011	0.612
PANSS total score	0.008	0.228	−0.013	0.113	0.003	0.649
CPZeq^a^ (mg/day)	−0.001	0.006 ^d^	0.001	0.699	−0.001	0.031 ^d^
BPDeq^b^ (mg/day)	0.045	0.512	−0.012	0.885	0.039	0.556
DZPeq^c^ (mg/day)	0.004	0.667	0.000	0.972	0.000	0.997
Type of *CYP1A2* rs762551 genotype (reference category: A allele carrier)	0.472	0.062	0.509	0.095	0.456	0.062
Type of *UGT1A4* rs2011425 genotype (reference category: G allele carrier)	−0.705	0.008 ^d^	0.324	0.302	−.369	0.143

*ANS* Autonomic nervous system, *BMI* Body mass index, *ln* natural log-transformed, *HF* High frequency, *LF* Low frequency, *PANSS* Positive and Negative Syndrome Scale, *TP* Total power

^a^The daily dosages of antipsychotic drugs were converted to approximate chlorpromazine equivalents

^b^The daily dosages of anticholinergic antiparkinsonian drugs were converted to approximate biperiden equivalents

^c^The daily dosages of benzodiazepine were converted to approximate diazepam equivalents

^d^Significant difference (*P* < 0.05; multiple regression analysis)

**Table 5:** Summary of demography and medication differences between participants treated with olanzapine monotherapy bearing the rs2011425 non-G allele and G allele

	rs2011425 T/T	rs2011425 G/T, G/G	*P*
Age (years)	51.77 ± 15.26	44.33 ± 14.08	0.064
Sex (male/female)	7/11	32/41	0.704
Smoking (Nonsmoker/smoker)	17/1	67/6	0.704
BMI (kg/m^2^)	24.12 ± 4.53	23.60 ± 5.29	0.675
PANSS total score	72.30 ± 16.75	71.61 ± 12.85	0.871
Disease duration (years)	19.86 ± 13.31	17.83 ± 14.83	0.574
CPZeq^a^ (mg/day)	490.41 ± 245.61	494.44 ± 260.03	0.951
BPDeq^b^ (mg/day)	0.81 ± 1.86	0.67 ± 1.57	0.766
DZPeq^c^ (mg/day)	7.97 ± 14.30	6.19 ± 9.19	0.953

Data are presented as mean ± standard deviation values

*BMI* Body mass index, *PANSS* Positive and Negative Syndrome Scale

^a^The daily dosages of antipsychotic drugs were converted to approximate chlorpromazine equivalents

^b^The daily dosages of anticholinergic antiparkinsonian drugs were converted to approximate biperiden equivalents

^c^The daily dosages of benzodiazepine were converted to approximate diazepam equivalents

## Discussion

The present study is the first, to the best of our knowledge, to analyze the association of polymorphisms in *CYP1A2*, *UGT1A4*, and *ABCB1*―namely rs2069514, rs762551, rs2011425, rs1045642, rs1128503, rs2032582, and rs2235048―with ANS dysfunction and other side effects in schizophrenia patients treated with the atypical antipsychotic olanzapine. Understanding the effects of olanzapine on ANS activity enables the safer application of antipsychotics in schizophrenia treatment. In this research, we found a significantly decreased LF component of HRV in olanzapine-treated schizophrenia patients with non-G alleles of the *UGT1A4* polymorphism rs2011425. The multiple regression analysis revealed a significant association between *UGT1A4* rs2011425 genotype and LF component after consideration of other clinical factors. This finding indicates that *UGT1A4* rs2011425 G allele, relative to non-G allele, is associated with higher sympathetic nervous system activity in schizophrenia patients treated with olanzapine. While there was also a tendency for differences in LF and TP components that were associated with *CYP1A2* polymorphism rs762551, multiple regression analysis did not reveal an association of LF or TP with this polymorphism. Thus, we conclude that *CYP1A2* rs762551 was not associated with ANS activity under olanzapine therapy after consideration of several other clinical factors. Regarding other genetic polymorphisms, no associations were observed between olanzapine-related ANS dysfunction and *CYP1A2* polymorphism rs2069514 or *ABCB1* polymorphisms rs1045642, rs1128503, rs2032582, or rs2235048.

Among patients treated with olanzapine, no significant differences were observed in age, sex, smoking status, BMI, symptom severity (as assessed by PANSS), duration of illness, or medication between patients with the rs2011425 G allele and non-G allele. Additionally, LF, which mainly reflects sympathetic nervous system activity, was significantly associated with *UGT1A4* rs2011425 genotype according to multiple regression analysis, which also revealed the impact of age and the dosages of antipsychotic agents on sympathetic nervous system activity (Table 3). There were no significant differences in age and the dosages of antipsychotics between patients with the *UGT1A4* rs2011425 G allele and non-G allele (Table 5). Therefore, we suggest that *UGT1A4* rs2011425 polymorphism was associated with sympathetic nervous system activity after controlling for other clinical factors, including age and dosages of antipsychotics. We also strongly suggest that the *UGT1A4* rs2011425 G allele is significantly associated with higher sympathetic nervous system activity (i.e., LF HRV).

UGT1A4 is the enzyme that is mainly responsible for olanzapine glucuronidation [10]. Among-individual variation in UGT1A4 activity has been linked to genetic polymorphisms, including *UGT1A4* rs2011425, known as UGT1A4*3, which exhibits an in vitro association with higher glucuronidation activity [40]. In previous research, the UGT1A4*3 variant has been associated with higher glucuronidation of olanzapine and thus substantially elevated levels of glucuronidated metabolites [41]. Ghotbi et al. [16] have reported that the *UGT1A4* rs2011425 G variant was associated with 25% lower average plasma concentrations of olanzapine. We conclude that the *UGT1A4* rs2011425 G allele likely causes rapid metabolism of olanzapine, resulting in reduced plasma concentrations of olanzapine and decreased olanzapine side effects, such as ANS dysfunction. Previous research on lamotrigine, which is metabolized by UGT1A4, showed that individuals with the non-G allele had elevated lamotrigine serum levels as well as improved treatment responses relative to individuals with the UGT1A4*3 variant [42]. Similarly, we assert that the response of olanzapine is also associated with serum levels of olanzapine.

*UGT1A4* rs2011425 is located in exon 1, and the G allele at this locus causes a leucine to valine amino acid substitution (L48V). The *UGT1A4* rs2011425 G genetic variant has been shown to be associated with a substrate-dependent increase or decrease in glucuronidation activity [43]. For UGT1A4 rs2011425, the L48V amino acid substitution was also associated with decreased tigogenin clearance. However, the clearance of clozapine, which is very structurally similar to olanzapine, was twice as high in individuals with the UGT1A4 valine-48 enzyme variant as in individuals with the more common variant [43]. The UGT1A4 L48V amino acid substitution has been speculated as causing ethylene-group-dependent steric changes in the enzyme’s substrate-binding region [16]. Accordingly, we propose that *UGT1A4* rs2011425 alleles determine steric phenotypes of UGT1A4, thereby affecting the metabolism of the substrate olanzapine, which results in interindividual differences in side effects, such as the observed sympathetic activity dysfunction.

In the present study, *CYP1A2* rs2069514 and rs762551 and *ABCB1* rs1045642, rs1128503, rs2032582, and rs2235048 polymorphisms were not observed to be associated with ANS activity in olanzapine-treated schizophrenia patients. However, associations of *CYP1A2* rs2069514 and rs762551 and *ABCB1* rs1045642, rs1128503, rs2032582, and rs2235048 gene polymorphisms with olanzapine serum concentration have been reported by changing CYP1A2 or P-gp activity, respectively [33, 44]. On the other hand, Ghotbi et al. [16] have reported no association of *CYP1A2* and *ABCB1* genetic variants with plasma concentrations of olanzapine. Similarly, other studies have also reported that *CYP1A2* rs2069514 and rs762551 polymorphisms have no effect on the activities of CYP1A2 in Japanese patients [45] and that *CYP1A2* gene polymorphisms did not appear to be associated with adverse reactions to olanzapine [46]. Thus, the effect of these polymorphisms (i.e., *CYP1A2* rs2069514 and rs762551 and *ABCB1* rs1045642, rs1128503, rs2032582, and rs2235048) on the activities of CYP1A2 or P-gp and on adverse responses to olanzapine remain controversial. Our results agree with the studies mentioned above, which reported no association between *CYP1A2* or *ABCB1* polymorphisms and reactions to olanzapine. Future studies are needed to validate the effects of these gene polymorphisms.

These results have the potential to improve clinical treatment of schizophrenia; however, our study has some limitations. We were unable to assay the serum and cerebrospinal fluid concentrations of olanzapine. We did not investigate adherence levels using objective indicators, and adherence might affect the concentrations of olanzapine. Additionally, the determination of causal relationships was not possible under the cross-sectional design employed. Additionally, this study included only Japanese patients, and this limits the generalizability of our results. We did not investigate the effect of CYP2D6 on ANS activity, and research investigating the joint impact of genetic variation in *CYP1A2*, *UGT1A4*, and *CYP2D6* is an essential objective for any future work. We cannot exclude the possibility that the tendency of differences in age between patients with *UGT1A4* rs2011425 G and non-G alleles interacts with the effect of rs2011425 genotype. Lastly, because the sample size was relatively small, larger studies conducted with more patients are required to confirm these findings.

## Conclusions

The findings of this study suggest that while *UGT1A4* genetic polymorphisms do affect olanzapine-related sympathetic nervous system activity, polymorphisms in *CYP1A2* and *ABCB1* do not. We suggest that the *UGT1A4* rs2011425 G variant affects the tolerability of olanzapine and is less likely to be associated with olanzapine side effects, including sympathetic dysfunction in schizophrenia patients. Thus, clinicians should consider the potentially elevated risk of ANS dysfunction in schizophrenia patients with *UGT1A4* rs2011425 non-G alleles before beginning treatment with olanzapine monotherapy. Larger follow-up studies should be conducted to confirm the pharmacogenomics of olanzapine, especially with regard to side effects such as ANS activity in schizophrenia patients.

## Data Availability

The datasets used and analyzed during the current study are available from the corresponding author on reasonable request.

## Abbreviations

ABCB1: ATP-binding cassette transport sub-family B member 1
ANS: autonomic nervous system
BMI: body mass index
CPZ: the daily doses of the antipsychotic drugs were converted to an approximate chlorpromazine equivalent
CYP: cytochrome p450
ECG: electrocardiography
HF: high frequency
HRV: heart rate variability
LF: low frequency
ln: natural log-transformed
MDR1: multidrug resistance protein 1
PANSS: Positive and Negative Syndrome Scale
P-gp: P-glycoprotein
SNP: single nucleotide polymorphism
TP: total power
UGT: uridine 5′-diphospho-glucuronosyltransferase
